# Sudden-onset Hypoglycemia Following Fluid Replacement in a Patient with Dapagliflozin-induced Diabetic Ketoacidosis Without Prior Insulin Use: Case Report

**DOI:** 10.7759/cureus.5448

**Published:** 2019-08-21

**Authors:** Ghada Elshimy, Ricardo Correa

**Affiliations:** 1 Endocrinology, Diabetes and Metabolism, University of Arizona College of Medicine-Phoenix, Phoenix, USA

**Keywords:** sodium-glucose co transporter-2 (sglt2) inhibitors, metformin, hypoglycemia, diabetic ketoacidosis

## Abstract

Euglycemic diabetic ketoacidosis (DKA) is a known complication of sodium-glucose co-transporter 2 (SGLT-2) inhibitors that have been reported in the literature. The prevalence of this side effect is growing and the exact mechanism of action on why this happens is unknown. Hypoglycemia events are very rare in diabetic patients using SGLT-2 inhibitors and/or metformin when they have normal kidney function. We report a novel complication of hypoglycemia that occurred during the course of treatment of SGLT2 inhibitor-induced DKA in a patient with type 2 diabetes mellitus (T2DM) on the dapagliflozin-metformin combination.

## Introduction

Sodium-glucose co-transporter 2 (SGLT-2) inhibitors are the most recent class of antihyperglycemic agents approved by the US Food and Drug Administration (FDA) to treat type 2 diabetes mellitus (T2DM). SGLT-2 inhibitors inhibit renal glucose reabsorption by blocking the sodium-glucose transporter. Although introduced as adjuncts to standard treatment regimens, new guidelines suggest that SGLT-2 inhibitors can be used as monotherapy in patients with T2DM because they provide cardiovascular and nephrological protection. SGLT-2 inhibitors are available as both single-ingredient products and in combination with metformin and other diabetic medications [1-2]. Because treatment with these agents was found to increase the incidence of diabetic ketoacidosis (DKA), the FDA issued a warning in 2015 about a causal relationship between DKA and SGLT-2 inhibitors. Most patients with DKA induced by these agents have had mildly elevated or normal blood glucose concentrations (i.e., euglycemic DKA) [3-7]. Immediate cessation of the medication is required to treat DKA [6]. To our knowledge, hypoglycemia after fluid replacement without starting insulin in patients using SGLT-2 inhibitors has not been reported.

## Case presentation

A 28-year-old woman presented to the emergency department with sudden-onset abdominal pain. She also had experienced multiple episodes of non-bloody vomitus during the previous 24 hours. One year earlier, she was diagnosed with uncontrolled T2DM but refused to start insulin treatment. Two months before presentation, her hemoglobin A1C concentration was 12%, and she was started on treatment with a combination of dapagliflozin and metformin.

At the time of presentation to the emergency department, the patient was afebrile with a mild increase in blood pressure to 138/86 mmHg. She was also tachypneic and tachycardiac, with a respiratory rate of 25 breaths/minute, a regular pulse with a heart rate of 116 beats/minute, and normal oxygen saturation. Her weight was 99 kg and her height was 179 cm with a body mass index (BMI) of 31 kg/m^2^. Physical examination revealed an obese dehydrated woman in mild distress and with mild diffuse abdominal tenderness, but no other remarkable findings. Her blood glucose concentration on Accu-check was 252 mg/dl. Laboratory testing showed an initial blood glucose concentration of 268 mg/dl, an anion gap of 18 mmol/l, and a pH of venous blood gases of 7.18. In addition, her bicarbonate concentration was 9 mmol/dl, her blood urea nitrogen was 50 mg/dl with creatinine 1.7 mg/dl, her acetone was elevated, and her hemoglobin A1C was 14.3%. Urinalysis showed moderate ketones with + 3 glucose. Her beta-human chorionic gonadotropin concentration and septic workup were negative, her cardiac enzymes were normal, and electrocardiography showed no signs of ischemia.

Following confirmation of DKA, the patient was admitted to the intensive care unit (ICU) and administered two liters of normal saline. Accu-check one hour later showed a blood glucose level of 55 mg/dl, a finding confirmed by a serum blood glucose concentration of 68 mg/dl, a significant reduction from the initial concentration of 268 mg/dl. The patient was administered 50 ml of a 50% dextrose solution; once her blood glucose stabilized, she was started on continuous insulin infusion along with the dextrose-containing intravenous solution. She was not treated with the dapagliflozin- metformin combination while in hospital. Her acidosis and anion gap were corrected over the following three days; because her hemoglobin A1C level remained elevated, she was discharged on the insulin regimen.

## Discussion

Fluid replacement was first reported important in a 1973 study of 37 young diabetic patients with severe euglycemic DKA [8]. More medications have become available for the treatment of patients with diabetes, with one class of agents, SGLT-2 inhibitors, reported to contribute to the occurrence of DKA [1-7]. Prospective randomized trials have shown that SGLT-2 inhibitors have multiple beneficial effects in diabetic patients. These include both cardiovascular and renoprotective effects, such as reducing blood pressure and body weight and improving renal hyperfiltration. SGLT-2 inhibitors also have anti-inflammatory effects through mechanisms that include weight loss and reduction of adipose tissue inflammation. SGLT-2 inhibitors also induce slight increases in ketone bodies and attenuate oxidative stress. Despite these benefits, SGLT-2 inhibitors carry considerable risk, as they predispose to urinary tract infection, Fournier’s gangrene, and DKA [9].

Volume replacement in patients with DKA has a significant impact on survival and in correcting hyperglycemia [8-10]. Rehydration was shown to improve the metabolic situation in patients with severe diabetic hyperglycemia and ketoacidosis by reducing the availability of counterregulatory hormones and by decreasing peripheral insulin resistance at a cellular level [10].

A review of 85 articles published between 1973 and 2016 on the management of DKA described the benefits of intensive hydration in these patients [10]. This analysis emphasized the importance of avoiding hypoperfusion and correcting marked hyperglycemia and hyperosmolarity. Other benefits include improving responses to insulin therapy. In 2009, the American Diabetes Association recommended that patients with DKA be infused with normal saline at a rate of 15 to 20 ml/kg body weight. An acceptable alternative is to infuse 1 to 1.5 liters normal saline during the first hour, with adjustments for any risk of cardiac compromise [11], with subsequent rates of intravenous fluid infusion depending on patient hemodynamic and hydration status and on serum electrolytes [11-12]. A lower rate is used in the pediatric population, 10 ml/kg body weight, to avoid the sudden drop of blood glucose [13]. Neither studies nor guidelines have addressed fluid replacement in obese patients with DKA.

This report describes a new potential complication that can occur during the early management of SGLT-2 inhibitor-induced DKA. DKA in our patient was induced by treatment with the combination of dapagliflozin and metformin. Despite having a high hemoglobin A1C concentration, the blood glucose in this patient on admission was not significantly elevated, which had led to hypoglycemia only after fluid replacement without insulin treatment. This complication may be unnoticed if the blood glucose concentration is not measured prior to starting insulin. The mechanism underlying hypoglycemia is not well understood. Our results suggest that in patients with DKA induced by SGLT-2 inhibitors, the rapid replacement of fluid with normal saline along with mildly elevated blood glucose can dramatically dilute extracellular glucose, resulting in hypoglycemia. In this type of scenario, we recommend regular monitoring of blood glucose and following the normal protocol used to treat DKA, consisting of replacement with 5% dextrose in patients with blood glucose concentrations <250 mg/dl. In addition, we suggest using the pediatric DKA protocol for fluid resuscitation in SGLT2 inhibitor-induced euglycemic DKA, which is 10 ml/kg instead of 15-20 ml/kg to avoid hypoglycemia. Our limitation is that this is a case report and further studies are required.

## Conclusions

Although SGLT-2 inhibitors are effective glucose-lowering agents and are prescribed as adjunctive or monotherapy for T2DM, it can have rare but dangerous potential adverse effects. Hypoglycemia after fluid replacement may be avoided in patients with SGLT-2 inhibitor-induced DKA by using a lower infusion rate of 10ml/kg for the initial fluid resuscitation, immediate blood glucose measurement post fluid resuscitation, and replacement with 5% dextrose in conjunction with starting insulin therapy.
